# Wild Emmer (*Triticum turgidum* ssp. *dicoccoides*) Diversity in Southern Turkey: Evaluation of SSR and Morphological Variations

**DOI:** 10.3390/life15020203

**Published:** 2025-01-29

**Authors:** Esra Çakır, Ahmad Alsaleh, Harun Bektas, Hakan Özkan

**Affiliations:** 1 Department of Field Crops, Faculty of Agriculture, Çukurova University, 01330 Adana, Turkey; 2 Department of Food and Agriculture, Institute of Hemp Research, Yozgat Bozok University, 66100 Yozgat, Turkey; ahmad.alsaleh@bozok.edu.tr; 3 Department of Agricultural Biotechnology, Faculty of Agriculture, Siirt University, 56100 Siirt, Turkey; bektasharun@gmail.com

**Keywords:** wild emmer, *T. turgidum* ssp. *dicoccoides*, SSR, plant morphology, Turkey

## Abstract

Wild emmer wheat (*Triticum turgidum* ssp. *dicoccoides*) is the ancestral species of cultivated tetraploid wheat with BBAA genomes. Because of its full interfertility with domesticated emmer wheat, this wild species can serve as one of the most important genetic resources to improve durum and bread wheat. To clarify the magnitude of genetic diversity between and within populations of Turkish wild emmer wheat, 169 genotypes of ssp. *dicoccoides* selected from the 38 populations collected from the three sub-regions (East-1, West-1, and West-2) of the Southeast Anatolia Region of Turkey were molecularly and morphologically characterized. The populations showed significant variation in plant height, heading date, flag leaf area, spike length and number, spikelet, peduncle, lemma, palea, glume and anther lengths, glume hull thickness, anther width, and days to maturity. According to the results of nuclear-SSR analysis, the populations collected from the sub-regions East-1 and West-2 were the most genetically distant (0.539), while the populations collected from the sub-regions West-1 and West-2 were the most genetically similar (0.788) populations. According to the results of AMOVA, there was 84% similarity within the populations studied, while the variation between the populations of the three sub-regions was 16%. In the dendrogram obtained by using nuclear-SSR data, the populations formed two main groups. The populations from the sub-region East-1 were in the first group, and the populations from the sub-regions West-1 and West-2 were in the second group. From the dendrogram, it appears that the populations from the sub-region East-1 were genetically distant from the populations from the sub-regions West-1 and West-2. The results highlight the potential diversity in Southeast Anatolia for wild emmer discovery and utilization.

## 1. Introduction

Crop wild relatives are the primary sources of novel genetic diversity that is needed for crop improvement in all aspects [1]. A comprehensive evaluation of in situ and ex situ resources, as well as common cultivars, to mine new alleles is a continuously needed process for crop improvement and agricultural sustainability [2]. The utilization of genetic resources not only requires a clear understanding of the allelic constitution of such species [3] but also requires the successful introduction of this diversity into the domesticated gene pool [4].

Wild emmer wheat (*Triticum turgidum* ssp. *dicoccoides*), as one of the closest known wild relatives of the domesticated tetraploid wheat, holds significant potential for wheat improvement [5,6,7]. It is the most likely ancestral species of cultivated tetraploid wheat with a genome constitution of BBAA. The geographical distribution area of wild emmer covers the Fertile Crescent in South-west Asia, from Israel, Jordan, Lebanon, Syria, southern Turkey, and northern Iraq to south/southwest Iran [8,9]. Due to its full interfertility with domesticated emmer wheat (*T. turgidum* subsp. *dicoccum* (Schrank ex Schübl.) Thell.), it can serve as one of the most important genetic resources to improve durum (*Triticum turgidum* L. ssp. *durum* (Desf.) and bread wheat (*Triticum aestivum* L.). Wild emmer has been used for allele mining for many needs of wheat breeding, including, but not limited to, drought [10,11] and salinity tolerance [5,12], and for biotic stress factors such as fusarium head blight [13,14], stripe rust [15,16], root-lesion nematodes [17], and powdery mildew [18,19,20,21,22]. In addition to stress tolerance and resistance-related introductions from the wild emmer genome, there has been extensive usage of wild emmer in many aspects of wheat improvement, including vernalization genes [23], drought-related traits [24], avenin-like proteins [25], and many others [6,8,11,24,26]. However, there is a need for cautious assessment because of the risk of introduction of undesirable genes [27,28,29,30].

Due to its possible domestication in or near the Levant [6,9], there has been a high level of reports from Israel and the vicinity in the Jordan Valley [31,32,33]. There are only a few studies on the accessions of wild emmer wheat from some locations out of Turkey [9,34,35,36], but there is not any comprehensive evaluation of in situ or ex situ gene pools from Southeast Anatolia. On the other hand, since the early 20th century, there has been a significant number of reports on the diversity and distribution of wild emmer wheat from the Levant and Jordan Valley, mostly Israel and Lebanon [31,32,37,38,39,40,41,42,43,44,45,46,47]. This concentrated evaluation of a specific region for wild emmer continued in the following years [7,11,16,20,48,49,50,51,52,53,54,55,56,57,58,59,60]. The evaluation of the natural populations in the central Fertile Crescent, especially Southeast Anatolia, is neglected. Even though this region has recently been emphasized for its role in wheat domestication [9,61], there is no comprehensive report of the natural wild emmer diversity in this region and Turkey. Therefore, the purpose of this study was to document the phenotypic and genotypic diversity between and within 169 accessions of 38 in situ populations of wild emmer wheat collected from Southeast Anatolia.

## 2. Materials and Methods

In this study, 169 accessions of 38 wild emmer wheat populations recently collected from Southeast Anatolia were used (Table 1). A total of 17 populations were collected from the Karadağ region (8 populations from Karadağ/West1 and 9 populations from Karadağ/West2 regions), near Gaziantep, while rest of the populations (21 populations from Karacadağ/East) were collected from the Karacadağ region, 200 km east, near Diyarbakır, Turkey. This study was conducted in Adana, Turkey during the 2010–2011 growing season. The seeds from each population were grown at the experimental area of Çukurova University, Adana, Turkey, to study genotypic and phenotypic variation. Initially, ten seeds from each genotype were germinated in Petri dishes. Once the seedlings developed, they were transferred to small pots. Each seedling was then transplanted into a separate row, maintaining a spacing of 30 cm between and within the rows. To prevent damage from spike breakage and to ensure complete self-pollination while preserving seed purity, the main spikes of all wild emmer plants (*Triticum turgidum* subsp. *dicoccoides*) were bagged for self-pollination.

Total genomic DNA was isolated from young leaves according to the cetyltrimethylammonium bromide (CTAB) protocol [62] with some modifications reported by Ozkan et al. [63]. The extracted DNA was evaluated qualitatively in addition to quantitively, and measured by 0.8% agarose gel electrophoresis. Before using DNA for molecular analysis, the DNA was diluted to the required concentration of 10 ng/mL for SSR applications. Initially, 100 SSR primers mapped to the A and B genomes were first screened on eight wild emmer genotypes not only to detect their polymorphism level but also PCR amplification. After the screening, 16 SSR primers were selected for further work (Table 2). M13 tailed-primer PCR amplification of SSRs according to [64] was performed in a 12 µL PCR mix containing 1X buffer, 0.125 mM dNTPs, 0.4 pmol M13 sequences tailed forward primer, 0.3 pmol reverse primers, 3.0 pmol universal M13 primer labeled with one of four (6-FAM, VIC, NED or PET) fluorescent dyes, 0.12U *Taq* DNA polymerase, and approximately 50 ng genomic DNA. PCR amplification was performed with an initial denaturation at 94 °C for 5 min; 30 cycles of 94 °C for 1 min, 55 to 67 °C (annealing temperature depending on primers) for 1 min, and 72 °C for 1 min; followed by 8 cycles of 94 °C for 30 s, 53 °C for 45 s, and 72 °C for 45 s; and a final extension at 72 °C for 10 min. A set of four PCR products (1 μL each) labeled with a different dye was combined with 0.25 μL GeneScan-400 LIZ^®^ size standards (Applied Biosystems, USA) and 9.86 µL Hi-Di™ Formamide (Applied Biosystems), denatured at 94 °C for 5 min, chilled on ice, and separated on an ABI 3130xl Genetic Analyzer (Applied Biosystems, USA). The SSR fragments were scored and checked twice using the Gene Mapper software v3.7 (Applied Biosystems, USA) as described in the user manual.

The SSR was scored as binary data (1/0), indicating the presence or absence of a marker in the genomic representation of each sample. Genetic distance was calculated using DARWin 6.0.13. Subsequently, these genetic distance data were used to construct a phylogenetic tree using the Neighbor-joining method in the MEGA11 software program. Several genetic diversity parameters were calculated for each locus and population using the GENALEX6.5 program [65]. Principal coordinate analysis (PCoA), used to explore multivariate relationships among inter-individual genetic distances within and among populations, was also performed with the GENALEX6.5 program.

## 3. Results

### 3.1. Agromorphological Variation in Wild Emmer Populations

A total of 169 accessions, collected as 38 populations from Southeast Anatolia, were evaluated for agromorphological and genetic-diversity-related traits. According to the variance analysis (ANOVA) based on three groups, there were significant (*p* < 0.05) differences between the evaluated panel of accessions for plant height, heading date, flag leaf area, spike length and number, spikelet, peduncle, lemma, palea, glume, and anther lengths, glume hull thickness, anther width, and days to maturity (Table 3).

Out of the 23 agromorphological parameters evaluated, the populations from Karacadag/EAST had the highest values in spike length (9.28 cm), spikelet number (20.69), glume hull thickness (0.26 mm), and anther width (0.59 mm). On the other hand, populations from the Karadag-1/WEST region had the highest agromorphological trait values in heading date (170.38 days), flag leaf area (17.76 cm^2^), auriculas width (5.68 mm), length of the uppermost awn in the spikelet (68.41 mm), spikelet length (16.13 mm), lemma length (13.95 mm), and palea length (12.88 mm). Finally, the populations from Karadag-2/WEST region had the highest values in plant height (128.31 cm), heading date (167.49 days), peduncle length (41.48 cm), auriculas length (4.65 mm), lemma width (2.62 mm), palea width (2.03 mm), glume length (12.61 mm), glume height (2.55 mm), anther length (4.10 mm), and maturation day (199.86 days). According to overall data, populations from the Karadag-2/WEST region had the maximum agromorphological diversity and the highest values in phenotypical traits (Table 4).

### 3.2. Genetic Diversity Within and Between the Populations and Sub-Regions

The genetic diversity of the evaluated accessions was assessed with analysis of molecular variance (AMOVA), and several diversity parameters including Nei genetic distance and identity values (Table 5 and Table 6). AMOVA was calculated to assess the variance within and between 38 populations of wild emmer wheat collected from three sub-regions of Southeast Anatolia (Table 5). There was a significant level of variation within the populations (84%), while the variation among the populations was 16%. Nei genetic distance and identity values for the three sub-regions are given in Table 5. According to the results, the regions with the most distance were Karacadag/EAST and Karadag-2/WEST (0.539), while the ones with the closest identity values were Karadag-1/WEST and Karadag-2/WEST (0.788). The lowest distance was seen between two Karacadağ west sub-regions (0.214), and the lowest identity was between Karacadag/EAST and Karadag-2/WEST regions (0.463).

We also calculated several genetic diversity parameters to estimate the variation within and between the populations. These parameters were the number of alleles per locus (Na), the number of effective alleles per locus (Ne), Shannon’s information index (I), expected heterozygosity (He), and unbiased heterozygosity (uHe). The highest number of alleles per locus was in the populations from the Karacadag/EAST region, while the lowest was in the populations from the Karadag-1/WEST region. Similarly, the populations from Karacadag/EAST region had the highest values in Ne, I, He, and uHe compared to the Karadag-1/WEST and Karadag-2/WEST populations (Table 7). In contrast, the lowest values in all parameters were obtained in the populations from the Karadag-1/WEST region. According to the above results, the maximum and minimum genetic diversity were obtained in the populations from Karacadag/EAST and Karadag-1/WEST regions, respectively.

### 3.3. PCoA and Neighbor-Joining Grouping Patterns in Wild Emmer Populations

**Figure 1:**
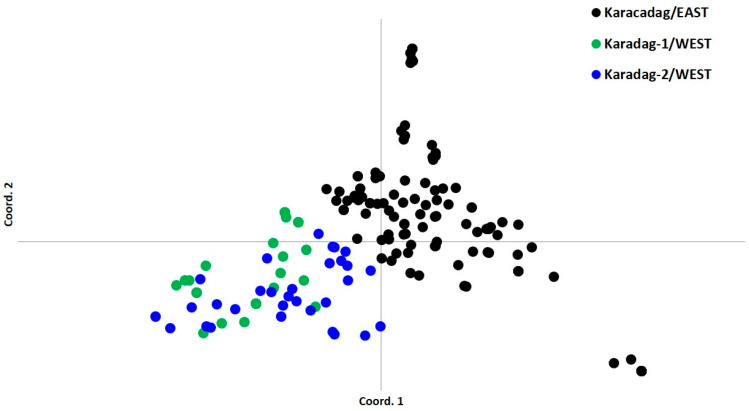
PCoA clustering of 38 *Triticum turgidum* ssp. *dicoccoides* populations collected from the Karacadağ region of Southeast Anatolia, central Fertile Crescent. Populations were Karacadag/EAST, Karadag-1/WEST, and Karadag-2/WEST.

The PCoA was applied to define the population interactions, and 169 accessions from 38 populations were separated into two major clusters without any mixture between Karacadag/EAST and both Karadag-1/WEST and Karadag-2/WEST regions. The distinction between East and West was quite sharp and there was no mixture (Figure 1) between these regions. Two sub-sets of Karadağ/WEST (1 and 2) were almost entirely mixed. So, it was not possible to separate them further into smaller sub-sets. Of the sub-cluster in Karacadag/EAST, there were several accessions located far away from the rest of the accessions.

**Figure 2:**
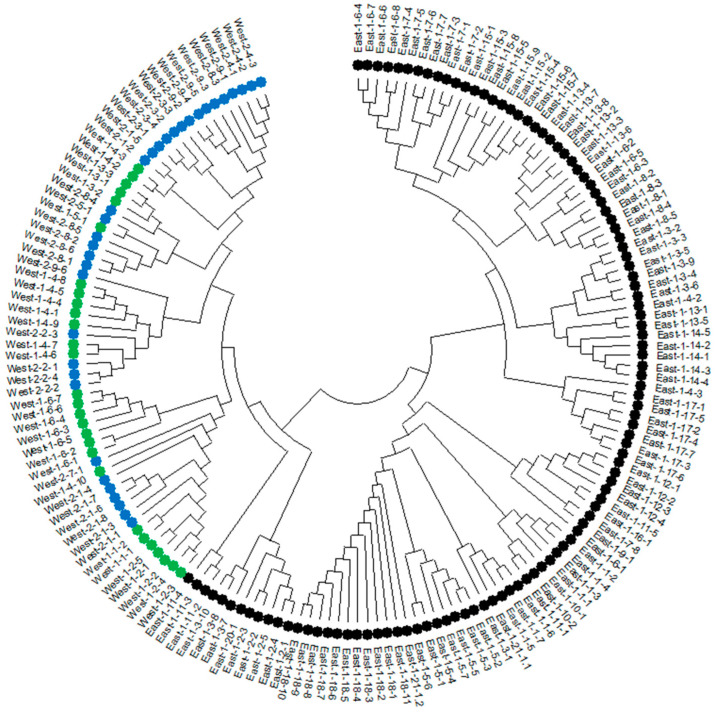
Neighbor-joining analysis was performed on 38 *Triticum turgidum* ssp. *dicoccoides* populations, comprising 169 accessions collected from Southeast Anatolia, within the central Fertile Crescent. The image uses different colors to represent these populations: black for Karacadag/EAST, green for Karadag-1/WEST, and blue for Karadag-2/WEST.

The relationships within and between the three regional groups were also evaluated using Neighbor-joining analysis (Figure 2). Neighbor-joining tree dendrograms produced a similar distribution to PCoA clustering. Two main branches were formed with accessions from WEST and EAST. These two main clusters did not have any admixture. There were several sub-clusters on the main EAST and WEST clusters. There were several sub-clusters in the main WEST cluster, which were almost entirely constituted from the mixed accessions of WEST-1 and WEST-2. There were only a few sub-clusters (WEST-uppermost branch) with a clear divergence from the rest of the group. Even though there were mixtures over the entire main WEST cluster, each small sub-set was formed with at least several accessions from the same sub-region.

## 4. Discussion

Crop wild relatives are one of the main sources of allelic diversity and thus the hub for climate-resilient crop breeding. To “feed the billions” [66] and meet the pace of climate change and population increase, there is a continuous need for allele mining and germplasm screening [67,68,69,70,71]. Wild emmer wheat, one of the closest relatives of durum wheat [72], is a possible shortcut to the wide wild allelic gene pool in *Triticum* sp. [8,73,74,75]. As highlighted, there is a need for a “walk on the wild side” [68]. With this objective in mind, we characterized a set of in situ germplasm accessions from Southeast Anatolia, the home of Göbeklitepe, the oldest known temple, for genetic diversity assessment [76,77].

### 4.1. Agromorphological Diversity

As a result of germplasm collection from the three different sub-regions in Southeast Anatolia, 169 accessions within 38 populations were characterized. Agromorphological characterization and genetic variation assessment through SSR markers were utilized to estimate the natural population diversity of this region. Wild emmer is thought to be domesticated near the southern Levant [6,9], and there has been a growing intensity in the number of reports from the possible domestication center. However, it has a much wider species distribution [6,8,9] and the number of reports from other regions (Turkey and Iran) is quite limited [34,35,78]. In addition, none of the previous reports from Turkey built an in-depth agromorphological and/or genetic diversity evaluation among the natural (in situ) populations.

Here, we obtained significant agromorphological and genetic diversity among and within the subsets of wild emmer wheat collected (Table 3 and Table 4). The main components of the traits we evaluated were spike, plant growth, and phenological traits.

Even though there is a vast number of studies in relation to biotic stress tolerance, such as powdery mildew [20,79], fusarium head blight [13], and stripe rust [15] in wild emmer, we did not find any reports concerning spike and phenology traits within this region. The results highlight the potential of in situ populations and hidden gems in the wild [80] and show that several spike-related traits such as purple coleoptile, purple auricle, purple culm, hairy auricle, hairy rachilla, and the fragility of the spike were controlled by single dominant genes, making the transfer of such traits much more straightforward compared to quantitatively inherited traits. Further studies should try to utilize germplasm resources with novel allelic diversity in the common crops as candidates for abiotic and biotic stress tolerance, as well as yield- and growth-related traits [13,20,25,79,81].

### 4.2. Genetic Diversity of In situ Populations

To evaluate the genetic diversity within and between the populations, 16 specific SSR markers were applied (Table 2). According to the results of AMOVA and other genetic diversity parameters, there was significant diversity among the evaluated panel of in situ accession. There were 84% within- and 16% between-population diversity. The results were in a similar range to previous reports in tetraploid wheat. In similar studies, Negisho et al. [82] reported 19 and 81% within- and between-population diversity, while Teklu et al. [83] reported wider levels of diversity among 73 wild emmer accessions from 11 different geographic regions. Nei genetic distance values in this study ranged between 0.214 (between Karadag-1/WEST and Karadag-2/WEST) and 0.539 (between Karacadag/EAST and Karadag-2/WEST). The results showed a clear-cut separation between the EAST and WEST populations. The mountainous landscape of the region seems to reduce genetic drift and mixture even within a relatively close distance of about 200 km. The distance between the WEST-1 and WEST-2 populations was small, about 55 km, and their genetic distance was quite small, compared to the WEST–EAST distance (Table 5). According to Harlan and Zohary [84], Zohary and Hopf [85], and Ozkan et al. [9], wild emmer has a wide distribution from the Levant to Turkey and south/southwest Iran. The level of observed diversity between the Karadağ and the Karacadağ regions demonstrates the potential of this unexplored habitat for crop improvement [6,11,86]. When we examined the diversity for climate (sub-Mediterranean to inner dry climates), elevation (650 m to 1300 m), mountainous landscapes, and slope, we discovered that geological and morphological diversity may be influenced by these characteristics. A rapidly changing climate and other geographical conditions may have resulted in unique population developments in this relatively small study area.

The number of alleles per locus (Na) is an indicator of the diversity at the gene level [87]. Here, we observed a three-fold difference between three regional groups from 3.93 in Karadag-1/WEST to 9.93 in Karacadag/EAST. Since all accessions were from the same species within the same wider region, this level of difference may be due to unequal population size distribution among sub-sets or some other unknown reasons. Li et al. [51] evaluated the number of microsatellites among 105 individuals from the Yehudiyya region of Israel; the Na values they obtained were lower than those in the current study. In their follow-up study, the same group obtained 1.88, 3.86, and 5.89 Na values at the chromosome, genome, and genome × chromosome levels. In a similar study, on a different region, Li et al. [47] reported an average 7.1 Na value using 28 microsatellite markers among 155 individuals from two sub-regions of Tabigha, Israel. In our study, the number of effective alleles (Ne) followed the same trend with the Na values, which ranged between 2.75 and 5.50 per locus. The Ne values were higher than those of Arystanbekkyzy et al. [34], who reported an average 1.962 Ne value among 29 wild and 4 cultivated emmer wheat populations collected from different regions of Turkey.

We obtained a Shannon index (I) between 1.030 (Karadag-1/WEST) and 1.692 (Karacadag/EAST). Expected heterozygosity (He) was between 0.561 and 0.725. The I and He values we obtained were significantly higher compared to those of Dong et al. [7] and Fahima et al. [45], while these were in similar ranges compared to those of Fahima et al. [50]. A similar study [36] compared accessions from Israel and Turkey (Diyarbakır region) for genetic diversity using AFLP markers. The He values they reported were much lower compared to those of the current study. Ozbek et al. [88] evaluated a set of accessions (120) from Israel and reported lower He values compared to our results. Here, the Na, Ne, I, He, and uHe values we obtained show the genetic diversity of the natural wild emmer populations in the Karacadağ region.

### 4.3. PCoA and Neighbor-Joining Analysis

PCoA and neighbor-joining trees followed the same trend (Figure 1 and Figure 2). Both methods separated WEST and EAST populations and did not create any mixture zones. On the other hand, two sub-sets of WEST (1 and 2) were almost entirely mixed and it was not possible to make a distinction between these two. Neighbor-joining dendrograms and PCoA clustering were similar to our previous report with AFLP markers [9], which distributed EAST and WEST populations in two separate clusters. When we looked closely at the neighbor-joining dendrogram for branching and genotype positions (Figure 2), it was seen that most of the individuals from the specific populations were located closely on the same branch, with some exceptions that did not follow any trend.

## 5. Conclusions

Wild and domesticated emmer wheat are well-characterized and excessively utilized species for crop improvement, especially in biotic stress tolerance. Here, a panel of wild emmer wheat was characterized for agromorphological traits and genetic diversity. According to the genetic diversity values obtained, analyzed through PCoA and neighbor-joining dendrograms, two regional groups with a distance of approximately 200 km had significantly different characteristics in terms of allelic distribution and some phenotypic traits. The screening and utilization of in situ germplasm sources from this region would help widen the genetic diversity in durum and common wheat breeding.

## Figures and Tables

**Table 1:** Collection site information about 38 wild emmer wheat (*Triticum turgidum* ssp. *dicoccoides*) populations.

Collection No	Collection Locality	Zone	Altitude (m)	Latitude (°N)	Longitude (°E)
1	24.5 km SW from Diyarbakır to Ovadag	East1	780	37°47′38″	40°12′14″
2	12.9 km NW from Ovadag to Pirinçlik	East1	1007	37°47′31″	39°57′18″
3	18.5 km NW from Ovadag to Pirinçlik	East1	920	37°49′17″	39°59′34″
4	20.1 km SW from Pirinçlik	East1	1080	37°52′02″	39°51′05″
5	20 km SW from Pirinçlik	East1	1260	37°50′40″	39°47′58″
6	2.9 km NE from Karabahçe to Pirinçlik	East1	1300	37°49′12″	39°46′29″
7	41.2 km SW from Pirinçlik	East1	1250	37°46′42″	39°44′50″
8	6.3 km N from Karabahçe (42.9 km W from Diyarbakır to Siverek)	East1	1070	37°50′21″	39°43′23″
9	4.6 km SW from Karabahçe	East1	1180	37°46′19″	39°44′03″
10	17.9 km SW from Karabahçe	East1	1160	37°44′29″	39°42′50″
11	21.7 km SW from Karabahçe	East1	1235	37°42′51″	39°44′03″
12	37.9 km SW from Karabahçe	East1	1170	37°39′49″	39°42′49″
13	37.9 km SW from Karabahçe	East1	1180	37°36′27″	39°43′41″
14	41.6 km SW from Karabahçe	East1	1170	37°35′08″	39°44′36″
15	48.7 km SW from Karabahçe	East1	1030	37°33′09″	39°42′06″
16	27.6 km SW from Karacadag (69.6 km SW from Karabahçe)	East1	950	37°37′40″	39°33′40″
17	30.2 km SW from Çermik to Siverek	East1	800	38°00′56″	39°22′11″
18	Siverek Karakeçi Road Azemi Village	East1	733	37°36′51″	39°20′12″
19	Karakeçi road	East1	737	37°33′27″	39°20′35″
20	Karakeçi grassland	East1	758	37°32′22″	39°21′52″
21	5 km from Siverek to Siverek Hilvan Road	East1	645	37°42′23″	39°16′34″
22	72 km SE from Turkoglu SE (W of Karadag)	West1	800(853)	37°19′46″	37°16′29″
23	72 km SE from Turkoglu SE (W of Karadag)	West1	800(853)	37°19′46″	37°16′29″
24	34 km ESE from Narlı (WSW of Karadag)	West1	840 (877)	37°18′53″	37°15′41″
25	34 km ESE from Narlı (WSW of Karadag)	West1	780 (813)	37°20′12″	37°17′53″
26	39 km ESE from Narlı (SW of Karadag)	West1	760 (793)	37°17′06″	37°17′39″
27	39 km ESE from Narlı (SW of Karadag)	West1	760 (793)	37°17′06″	37°17′39″
28	Between Kahramanmaraş Kelleş village and Yiğitce village	West1	791	37°20′25″	37°17′54″
29	Between Gaziantep Tekirsin village and Dundarlı village	West1	882	37°15′20″	37°23′26″
30	37 km NE from Kilis to Gaziantep	West2	830	37°20′19″	37°16′50″
31	39 km NE from Kilis to Gaziantep	West2	920	37°19′50″	37°18′51″
32	41 km NE from Kilis to Gaziantep	West2	770	37°24′23″	37°25′47″
33	42 km NE from Kilis to Gaziantep	West2	750	37°24′58″	37°24′50″
34	58 km NE from Kilis to Gaziantep	West2	720	37°16′01″	37°30′52″
35	59 km NE from Kilis to Gaziantep	West2	770	37°15′33″	37°29′03″
36	21 km NE from Kilis to Gaziantep	West2	620	36°45′52″	37°15′04″
37	24 km NE from Kilis to Gaziantep	West2	700	36°52′20″	37°12′12″
38	25 km NE from Kilis to Gaziantep	West2	830	36°33′25″	37°11′57″

**Table 2:** List of SSR primers used in this study with respective repeat motifs and chromosome locations.

**Name**	**Ch**	**Motif**	**Forward Primer Sequence**	**Reverse Primer Sequence**
cfa2219	1A	(GT)21	TCTGCCGAGTCACTTCATTG	GACAAGGCCAGTCCAAAAGA
wmc312	1A	(GA)10	TGTGCCCGCTGGTGCGAAG	CCGACGCAGGTGAGCGAAG
wmc658	2A	----	CTCATCGTCCTCCTCCACTTTG	GCCATCCGTTGACTTGAGGTTA
wmc313	4A	(CA)18	GCAGTCTAATTATCTGCTGGCG	GGGTCCTTGTCTACTCATGTCT
wmc110	5A	(GT)11	GCAGATGAGTTGAGTTGGATTG	GTACTTGGAAACTGTGTTTGGG
cfa2190	5A	(TC)31	CAGTCTGCAATCCACTTTGC	AAAAGGAAACTAAAGCGATGGA
wmc626	1B	----	AGCCCATAAACATCCAACACGG	AGGTGGGCTTGGTTACGCTCTC
gwm498	1B	----	GGTGGTATGGACTATGGACACT	GGTGGTATGGACTATGGACACT
wmc128	1B	(GA)10	CGGACAGCTACTGCTCTCCTTA	CTGTTGCTTGCTCTGCACCCTT
wmc149	2B	(CT)24	ACAGACTTGGTTGGTGCCGAGC	ATGGGCGGGGGTGTAGAGTTTG
wmc332	2B	(CT)12	CATTTACAAAGCGCATGAAGCC	GAAAACTTTGGGAACAAGAGCA
gwm335	5B	---	CGTACTCCACTCCACACGG	CGGTCCAAGTGCTACCTTTC
gwm630	6B	(GT)16	GTGCCTGTGCCATCGTC	CGAAAGTAACAGCGCAGTGA
gwm146	7B	---	CCAAAAAAACTGCCTGCATG	CTCTGGCATTGCTCCTTGG
wmc76	7B	(GT)19	CTTCAGAGCCTCTTTCTCTACA	CTGCTTCACTTGCTGATCTTTG
gwm333	7B	(GA)19	GCCCGGTCATGTAAAACG	TTTCAGTTTGCGTTAAGCTTTG

**Table 3:** Results of analysis of variance on studied traits.

Traits	Sum of Squares	Mean Square
Plant height	990.53	495.269 *
Heading date	750.785	375.393 ***
Peduncle length	1155.930	577.695 ***
Flag leaf area	262.141	131.071 ****
Auriculas length	0.927	0.463 ^ns^
Auriculas width	2444.0	1222.0 ^ns^
Spike length	5387.0	2694.0 *
Spike number	137.496	68.748 ****
Length of the uppermost awn in the spikelet	822.418	411.209 ^ns^
Awn length in the fourth flower	1474.411	737.205 ^ns^
Length of the lowermost awn in the spikelet	1236.780	618.390 ^ns^
Spikelet length	13.883	6941.0 *
Spikelet width	2467.0	1233.0 ^ns^
Lemma length	18.152	9076.0 **
Lemma width	0.263	0.132 ^ns^
Palea length	7100.0	3550.0 *
Palea width	0.239	0.119 ^ns^
Glume length	7473.0	3736.0 *
Glume hull thickness	6745.0	3373.0 *
Glume height	0.481	0.240 ^ns^
Anther length	4556.0	2278.0 ****
Anther width	0.068	0.034 *
Maturation	673.340	366.670 *

****, ***, **, * Significantly different at the *p* < 0.0001, *p* < 0.001, *p* < 0.01, and *p* < 0.05 levels, respectively. n.s. = not significant.

**Table 4:** Mean values of agromorphological traits of wild emmer wheat (*Triticum turgidum* ssp. *dicoccoides*) genotypes sampled from three different sub-regions of Southeast Anatolia.

Traits	Whole Collections	Karacadag/EAST	Karadag-1/WEST	Karadag-2/WEST
Plant height (cm)	126.95 ±18.45	126.35 ± 19.68	127.64 ± 16.27	128.31 ± 16.28
Heading date (day)	166.06 ± 8.36	164.37 ± 9.12	170.38 ± 7.08	167.49 ± 4.58
Peduncle length(cm)	38.95 ± 6.85	39.17 ± 6.83	35.09 ± 7.09	41.48 ± 5.26
Flag leaf area (cm^2^)	16.38 ± 6.18	15.66 ± 6.33	17.76 ± 5.76	17.55 ± 4.65
Auriculas length (mm)	4.52 ± 0.64	4.50 ± 0.68	4.45 ± 0.39	4.65 ± 0.65
Auriculas width(mm)	5.42 ± 0.90	5.43 ± 0.88	5.68 ± 0.87	5.19 ± 0.91
Spike length (cm)	9.18 ± 1.16	9.28 ± 1.23	9.11 ± 0.91	8.91 ± 1.08
Spikelet number	20.02 ± 2.49	20.69 ± 2.62	18.89 ± 1.75	18.80 ± 1.67
Length of the uppermost awn in the spikelet (mm)	76.37 ± 18.84	74.91 ± 19.56	80.76 ± 15.78	77.40 ± 18.71
Awn length in the fourth flower(mm)	95.97 ± 19.73	93.68 ± 20.45	100.23 ± 17.34	98.85 ± 18.51
Length of the lowermost awn in the spikelet (mm)	59.49 ± 22.21	56.35 ± 22.72	68.41 ± 18.75	62.11 ± 21.37
Spikelet length (mm)	15.72 ± 1.44	15.50 ± 1.47	16.13 ± 1.49	16.07 ± 1.17
Spikelet width (mm)	4.67 ± 0.82	4.74 ± 0.86	4.38 ± 0.68	4.71 ± 0.72
Lemma length (mm)	13.46 ± 1.66	13.26 ± 1.87	13.95 ± 1.30	13.71 ± 0.91
Lemma width (mm)	2.53 ± 0.35	2.50 ± 0.35	2.51 ± 0.35	2.62 ± 0.37
Palea length (mm)	12.49 ± 1.12	12.32 ± 1.19	12.88 ± 1.02	12.70 ± 0.86
Palea width (mm)	1.81 ± 0.51	1.77 ± 0.26	1.73 ± 0.26	2.03 ± 1.01
Glume length (mm)	12.27 ± 1.12	12.09 ± 1.14	12.59 ± 1.04	12.61 ± 1.04
Glume hull thickness (mm)	0.24 ± 0.09	0.26 ± 0.09	0.22 ± 0.09	0.20 ± 0.06
Glume height (mm)	2.45 ± 0.35	2.43 ± 0.33	2.45 ± 0.35	2.55 ± 0.40
Anther length (mm)	3.73 ± 0.50	3.62 ± 0.50	3.80 ± 0.43	4.10 ± 0.42
Anther width (mm)	0.58 ± 0.10	0.59 ± 0.10	0.53 ± 0.09	0.58 ± 0.11
Maturation (day)	198.12 ± 4.56	97.18 ± 4.97	199.48 ± 4.03	199.86 ± 2.48

**Table 5:** Analysis of molecular variance (AMOVA) summary for the variation within and between populations of *Triticum turgidum* ssp. *dicoccoides*.

Source	df	SS	MS	Est. Var.	%
Among Pops	2	439.551	219.776	4.429	16
Within Pops	166	3803.206	22.911	22.911	84
Total	168	4242.757		27.340	100

**Table 6:** Nei genetic distance (above diagonal) and Nei identity (below diagonal) values among 38 *Triticum turgidum* ssp. *dicoccoides* populations.

Population	Karacadag/EAST	Karadag-1/WEST	Karadag-2/WEST
Karacadag/EAST	---	0.518	0.539
Karadag-1/WEST	0.484	---	0.214
Karadag-2/WEST	0.463	0.788	---

**Table 7:** Summary statistics of genetic variation among three different regions, Karacadag/EAST, Karadag-1/WEST, and Karadag-2/WEST. The number of alleles per locus (Na), number of effective alleles per locus (Ne), Shannon’s information index (I), expected heterozygosity (He), and unbiased heterozygosity (uHe) of 38 *Triticum turgidum* ssp. *dicoccoides* populations.

Pops	N	Na	Ne	I	He	uHe
Karacadag/EAST	108	9.938 ± 1.871	5.470 ± 0.869	1.692 ± 0.177	0.725 ± 0.046	0.729± 0.046
Karadag-1/WEST	28	3.938 ± 0.470	2.747 ± 0.308	1.030 ± 0.118	0.561 ± 0.051	0.572 ± 0.052
Karadag-2/WEST	33	6.125 ± 0.861	4.135 ± 0.644	1.348 ± 0.181	0.621 ± 0.070	0.632 ± 0.071

## Data Availability

The original contributions presented in this study are included in this article. Further inquiries can be directed to the corresponding authors.
